# Effects of Autonomous Sensory Meridian Response (ASMR) on mental health.

**DOI:** 10.1192/j.eurpsy.2023.2045

**Published:** 2023-07-19

**Authors:** T. Gutierrez Higueras, A. Jiménez Peinado, B. Hernández Gajate, M. Reyes López, L. Montes Arjona, M. Rodriguez Ruzafa

**Affiliations:** 1 Psychiatry, Hospital Universitario Reina Sofía, Córdoba, Spain

## Abstract

**Introduction:**

The Autonomous Sensory Meridian (ASMR) is a static or tingling sensation on the skin that usually starts on the scalp and runs through the back of the neck and upper spine. It has been compared to tactile auditory synesthesia and may overlap with shivering. It is a subjective experience of “low-grade euphoria”, characterized by “a combination of positive feelings and a static tingling sensation on the skin”. It is most commonly triggered by auditory or visual stimuli, and less commonly by intentional attentional control.

**Objectives:**

To determine the effects produced by the perception of ASMR in the population with mental disorders.

**Methods:**

A literature review was carried out in Pubmed using the descriptors: “ASMR” AND “mental”. 7 results are obtained. The results of a time limit of 10 years were filtered, obtaining 6 results and selecting all of them for their relevance to the PICO question. Subsequently, the search was repeated using the same descriptors and time limit in the Cochrane Library and NICE, in which no results were found.

**Results:**

The first result, an RCT of 475 people between the ages of 18 and 54, showed that 80% of the participants answered positively when asked if ASMR has an effect on their mood, while 14% were not sure and 6 % felt ASMR did not alter their mood. When subjected to a mixed ANOVA with factors for time (before, during, immediately after, and 3 h after ASMR) and for depression status (high, medium, or low as defined by the BDI), we found a significant main effect. of time in mood. [p<0.0005]

In one of these studies, the default neural network (the one that works when the brain is relaxed) was analyzed in 11 volunteers in whom ASMR caused them to relax, in contrast to 11 individuals in the control group. At the end of the study, the ASMR volunteers generally showed less functional connectivity than the other volunteers. It also showed “increased connectivity between regions of the occipital, frontal, and temporal cortices,” suggesting that ASMR favors the association of those networks that are activated in the resting state.

**Conclusions:**

With the available evidence it is concluded that ASMR could improve of the affective clinic reflected in the parameters of the Beck depression scale as well as a sense of calm and relaxation and it reduces the heart rate or increases the conductivity of the skin, something that happens when certain emotional states are altered.

**Disclosure of Interest:**

None Declared

